# Supplementary material for: Memory retrieval effects as a function of differences in phenomenal experience
Source: Brain Imaging Behav. 2024 May 6;18(5):943–50. doi: 10.1007/s11682-024-00892-9 (PMC11582146; doi:10.1007/s11682-024-00892-9)
Supplement: Supplementary file 1 — Supplementary Material 1 [file 11682_2024_892_MOESM1_ESM.docx]

**Supplementary Materials**

*Main Effects of Stimulus*

|  |
| --- |
| Figure S1. Main effect of stimulus type during encoding (A) and retrieval (B). Warm (Yellow/Red) colors indicate greater activation (Hits – CRs) for faces (Faces > Words). Cool colors indicate greater activation for words (Words > Faces). |

*fMRIprep preprocessing pipeline*

Results included in this manuscript come from preprocessing performed using *FMRIPREP* version 20.2.1 [1, 2, RRID:SCR_016216], a Nipype [3, 4, RRID:SCR_002502] based tool. Each T1w (T1-weighted) volume was corrected for INU (intensity non-uniformity) using N4BiasFieldCorrection v2.1.0 [5] and skull-stripped using antsBrainExtraction.sh v2.1.0 (using the OASIS template). Brain surfaces were reconstructed using recon-all from FreeSurfer v6.0.1 [6, RRID:SCR_001847], and the brain mask estimated previously was refined with a custom variation of the method to reconcile ANTs-derived and FreeSurfer-derived segmentations of the cortical gray-matter of Mindboggle [21, RRID:SCR_002438]. Spatial normalization to the ICBM 152 Nonlinear Asymmetrical template version 2009c [7, RRID:SCR_008796] was performed through nonlinear registration with the antsRegistration tool of ANTs v2.1.0 [8, RRID:SCR_004757], using brain-extracted versions of both T1w volume and template. Brain tissue segmentation of cerebrospinal fluid (CSF), white-matter (WM) and gray-matter (GM) was performed on the brain-extracted T1w using fast [17] (FSL v5.0.9, RRID:SCR_002823).

Functional data was motion corrected using mcflirt (FSL v5.0.9 [9]). "Fieldmap-less" distortion correction was performed by co-registering the functional image to the same-subject T1w image with intensity inverted [13,14] constrained with an average fieldmap template [15], implemented with antsRegistration (ANTs). This was followed by co-registration to the corresponding T1w using boundary-based registration [16] with six degrees of freedom, using bbregister (FreeSurfer v6.0.1). Motion correcting transformations, field distortion correcting warp, BOLD-to-T1w transformation and T1w-to-template (MNI) warp were concatenated and applied in a single step using antsApplyTransforms (ANTs v2.1.0) using Lanczos interpolation.

Physiological noise regressors were extracted applying CompCor [18]. Principal components were estimated for the two CompCor variants: temporal (tCompCor) and anatomical (aCompCor). A mask to exclude signal with cortical origin was obtained by eroding the brain mask, ensuring it only contained subcortical structures. Six tCompCor components were then calculated including only the top 5% variable voxels within that subcortical mask. For aCompCor, six components were calculated within the intersection of the subcortical mask and the union of CSF and WM masks calculated in T1w space, after their projection to the native space of each functional run. Frame-wise displacement [19] was calculated for each functional run using the implementation of Nipype.

Many internal operations of FMRIPREP use Nilearn [22, RRID:SCR_001362], principally within the BOLD-processing workflow. For more details of the pipeline see <https://fmriprep.readthedocs.io/en/20.2.1/workflows.html>.

**Copyright Waiver**

The above boilerplate text was automatically generated by fMRIPrep with the express intention that users should copy and paste this text into their manuscripts *unchanged*. It is released under the [CC0](https://creativecommons.org/publicdomain/zero/1.0/) license.

**References**

1. Esteban O, Markiewicz CJ, Blair RW, Moodie CA, Isik AI, Erramuzpe A, Kent JD, Goncalves M, DuPre E, Snyder M, Oya H, Ghosh SS, Wright J, Durnez J, Poldrack RA, Gorgolewski KJ. fMRIPrep: a robust preprocessing pipeline for functional MRI. Nat Meth. 2018; doi:[10.1038/s41592-018-0235-4](https://doi.org/10.1038/s41592-018-0235-4)

2. fMRIPrep Available from: [10.5281/zenodo.852659](https://doi.org/10.5281/zenodo.852659).

3. Gorgolewski K, Burns CD, Madison C, Clark D, Halchenko YO, Waskom ML, Ghosh SS. Nipype: a flexible, lightweight and extensible neuroimaging data processing framework in python. Front Neuroinform. 2011 Aug 22;5(August):13. doi:[10.3389/fninf.2011.00013](https://doi.org/10.3389/fninf.2011.00013).

4. Gorgolewski KJ, Esteban O, Ellis DG, Notter MP, Ziegler E, Johnson H, Hamalainen C, Yvernault B, Burns C, Manhães-Savio A, Jarecka D, Markiewicz CJ, Salo T, Clark D, Waskom M, Wong J, Modat M, Dewey BE, Clark MG, Dayan M, Loney F, Madison C, Gramfort A, Keshavan A, Berleant S, Pinsard B, Goncalves M, Clark D, Cipollini B, Varoquaux G, Wassermann D, Rokem A, Halchenko YO, Forbes J, Moloney B, Malone IB, Hanke M, Mordom D, Buchanan C, Pauli WM, Huntenburg JM, Horea C, Schwartz Y, Tungaraza R, Iqbal S, Kleesiek J, Sikka S, Frohlich C, Kent J, Perez-Guevara M, Watanabe A, Welch D, Cumba C, Ginsburg D, Eshaghi A, Kastman E, Bougacha S, Blair R, Acland B, Gillman A, Schaefer A, Nichols BN, Giavasis S, Erickson D, Correa C, Ghayoor A, Küttner R, Haselgrove C, Zhou D, Craddock RC, Haehn D, Lampe L, Millman J, Lai J, Renfro M, Liu S, Stadler J, Glatard T, Kahn AE, Kong X-Z, Triplett W, Park A, McDermottroe C, Hallquist M, Poldrack R, Perkins LN, Noel M, Gerhard S, Salvatore J, Mertz F, Broderick W, Inati S, Hinds O, Brett M, Durnez J, Tambini A, Rothmei S, Andberg SK, Cooper G, Marina A, Mattfeld A, Urchs S, Sharp P, Matsubara K, Geisler D, Cheung B, Floren A, Nickson T, Pannetier N, Weinstein A, Dubois M, Arias J, Tarbert C, Schlamp K, Jordan K, Liem F, Saase V, Harms R, Khanuja R, Podranski K, Flandin G, Papadopoulos Orfanos D, Schwabacher I, McNamee D, Falkiewicz M, Pellman J, Linkersdörfer J, Varada J, Pérez-García F, Davison A, Shachnev D, Ghosh S. Nipype: a flexible, lightweight and extensible neuroimaging data processing framework in Python. 2017. doi:[10.5281/zenodo.581704](https://doi.org/10.5281/zenodo.581704).

5. Tustison NJ, Avants BB, Cook PA, Zheng Y, Egan A, Yushkevich PA, Gee JC. N4ITK: improved N3 bias correction. IEEE Trans Med Imaging. 2010 Jun;29(6):1310–20. doi:[10.1109/TMI.2010.2046908](https://doi.org/10.1109/TMI.2010.2046908).

6. Dale A, Fischl B, Sereno MI. Cortical Surface-Based Analysis: I. Segmentation and Surface Reconstruction. Neuroimage. 1999;9(2):179–94. doi:[10.1006/nimg.1998.0395](https://doi.org/10.1006/nimg.1998.0395).

7. Fonov VS, Evans AC, McKinstry RC, Almli CR, Collins DL. Unbiased nonlinear average age-appropriate brain templates from birth to adulthood. NeuroImage; Amsterdam. 2009 Jul 1;47:S102. doi:[10.1016/S1053-8119(09)70884-5](https://doi.org/10.1016/S1053-8119(09)70884-5).

8. Avants BB, Epstein CL, Grossman M, Gee JC. Symmetric diffeomorphic image registration with cross-correlation: evaluating automated labeling of elderly and neurodegenerative brain. Med Image Anal. 2008 Feb;12(1):26–41. doi:[10.1016/j.media.2007.06.004](https://doi.org/10.1016/j.media.2007.06.004).

9. Jenkinson M, Bannister P, Brady M, Smith S. Improved optimization for the robust and accurate linear registration and motion correction of brain images. Neuroimage. 2002 Oct;17(2):825–41. doi:[10.1006/nimg.2002.1132](https://doi.org/10.1006/nimg.2002.1132).

10. Andersson JLR, Skare S, Ashburner J. How to correct susceptibility distortions in spin-echo echo-planar images: application to diffusion tensor imaging. Neuroimage. 2003 Oct;20(2):870–88. doi:[10.1016/S1053-8119(03)00336-7](https://doi.org/10.1016/S1053-8119(03)00336-7).

11. Cox RW. AFNI: software for analysis and visualization of functional magnetic resonance neuroimages. Comput Biomed Res. 1996 Jun;29(3):162–73. doi:[10.1006/cbmr.1996.0014](https://doi.org/10.1006/cbmr.1996.0014).

12. Jenkinson M. Fast, automated, N-dimensional phase-unwrapping algorithm. Magn Reson Med. 2003 Jan;49(1):193–7. doi:[10.1002/mrm.10354](https://doi.org/10.1002/mrm.10354).

13. Huntenburg JM. Evaluating nonlinear coregistration of BOLD EPI and T1w images. Freie Universität Berlin; 2014. Available from: <http://hdl.handle.net/11858/00-001M-0000-002B-1CB5-A>.

14. Wang S, Peterson DJ, Gatenby JC, Li W, Grabowski TJ, Madhyastha TM. Evaluation of Field Map and Nonlinear Registration Methods for Correction of Susceptibility Artifacts in Diffusion MRI. Front Neuroinform. 2017 [cited 2017 Feb 21];11. doi:[10.3389/fninf.2017.00017](https://doi.org/10.3389/fninf.2017.00017).

15. Treiber JM, White NS, Steed TC, Bartsch H, Holland D, Farid N, McDonald CR, Carter BS, Dale AM, Chen CC. Characterization and Correction of Geometric Distortions in 814 Diffusion Weighted Images. PLoS One. 2016 Mar 30;11(3):e0152472. doi:[10.1371/journal.pone.0152472](https://doi.org/10.1371/journal.pone.0152472).

16. Greve DN, Fischl B. Accurate and robust brain image alignment using boundary-based registration. Neuroimage. 2009 Oct;48(1):63–72. doi:[10.1016/j.neuroimage.2009.06.060](https://doi.org/10.1016/j.neuroimage.2009.06.060).

17. Zhang Y, Brady M, Smith S. Segmentation of brain MR images through a hidden Markov random field model and the expectation-maximization algorithm. IEEE Trans Med Imaging. 2001 Jan;20(1):45–57. doi:[10.1109/42.906424](https://doi.org/10.1109/42.906424).

18. Behzadi Y, Restom K, Liau J, Liu TT. A component based noise correction method (CompCor) for BOLD and perfusion based fMRI. Neuroimage. 2007 Aug 1;37(1):90–101. doi:[10.1016/j.neuroimage.2007.04.042](https://doi.org/10.1016/j.neuroimage.2007.04.042).

19. Power JD, Mitra A, Laumann TO, Snyder AZ, Schlaggar BL, Petersen SE. Methods to detect, characterize, and remove motion artifact in resting state fMRI. Neuroimage. 2013 Aug 29;84:320–41. doi:[10.1016/j.neuroimage.2013.08.048](https://doi.org/10.1016/j.neuroimage.2013.08.048).

20. Pruim RHR, Mennes M, van Rooij D, Llera A, Buitelaar JK, Beckmann CF. ICA-AROMA: A robust ICA-based strategy for removing motion artifacts from fMRI data. Neuroimage. 2015 May 15;112:267–77. doi:[10.1016/j.neuroimage.2015.02.064](https://doi.org/10.1016/j.neuroimage.2015.02.064).

21. Klein A, Ghosh SS, Bao FS, Giard J, Häme Y, Stavsky E, et al. Mindboggling morphometry of human brains. PLoS Comput Biol 13(2): e1005350. 2017. doi:[10.1371/journal.pcbi.1005350](https://doi.org/10.1371/journal.pcbi.1005350).

22. Abraham A, Pedregosa F, Eickenberg M, Gervais P, Mueller A, Kossaifi J, Gramfort A, Thirion B, Varoquaux G. Machine learning for neuroimaging with scikit-learn. Front in Neuroinf 8:14. 2014. doi:[10.3389/fninf.2014.00014](https://doi.org/10.3389/fninf.2014.00014).
